# Initial Low-Density Lipoprotein Cholesterol and Inflammation Status Predicts Long-Term Mortality in Patients with Acute Coronary Syndrome in the Chinese Population

**DOI:** 10.3390/biomedicines13071534

**Published:** 2025-06-24

**Authors:** Yanqiao Lu, Yujun Sun, Yutong Miao, Zhitong Liu, Lan Shen, Ben He

**Affiliations:** 1 Department of Cardiology, Shanghai Chest Hospital, School of Medicine, Shanghai Jiao Tong University, Shanghai 200030, China; luyanqiao2013@live.com (Y.L.); yujunsun@icloud.com (Y.S.); miaoyutong8128@163.com (Y.M.); lzt_gdgz@sjtu.edu.cn (Z.L.); 2 Department of Cardiology, Shanghai Sixth People’s Hospital, School of Medicine, Shanghai Jiao Tong University, Shanghai 200233, China; 3 Cardiac Center, Shanghai Jiahui International Hospital, Shanghai 200000, China; 4 Medical Center, Harrisburg Hospital, University of Pittsburgh, Pittsburgh, PA 17050, USA

**Keywords:** acute coronary syndrome, hyperlipidemia, CRP, risk assessment, LDL-C

## Abstract

**Background**: Low-density lipoprotein cholesterol (LDL-C) is considered an important risk factor for acute coronary syndrome (ACS). Recent studies have revealed high mortality in ACS patients with low LDL-C levels. However, the association between spontaneously very low LDL-C levels and the prognosis in ACS remains unknown. **Methods**: A total of 1882 consecutive statin-null ACS patients were analyzed and categorized into four groups according to their on-admission LDL-C level: very low <70 mg/dL, low 70–99 mg/dL, high 100–129 mg/dL, and very high ≥130 mg/dL. In-hospital mortality and 3-year mortality were assessed. Among them, 1009 patients were further grouped according to the hs-CRP value (<2 mg/L and ≥2 mg/L). **Results**: Over one-third of the patients had an initially lower LDL-C concentration. Higher in-hospital mortality (9.7%, 4.5%, 2.7%, and 3.5%, *p* = 0.001), long-term mortality (20.8%, 13.1%, 8.0%, and 7.8%, *p* < 0.001), and lower survival rate (KM: HR = 3.15, 95% CI 1.40–7.12, *p* < 0.001; Cox: HR = 2.09, 95% CI 1.30 to 3.36) were observed in the very low LDL-C group compared with other groups. Patients in the low LDL-C high CRP subgroup had the worst prognosis compared with other subgroups (in-hospital: 7.7%, 1.2%, 0.5%, and 4.3%, *p* = 0.031; long-term: 15.5%, 1.2%, 2.6%, and 9.4%, *p* = 0.018). Lower LDL-C levels were accompanied by higher CRP levels (*p* = 0.003). The CRP–LDL-C ratio had good predictive ability on short-term and long-term outcomes (AUC: 0.630 and 0.738). **Conclusions**: Spontaneously very low LDL-C level was independently associated with poor long-term survival in patients with ACS. Lower LDL-C level was related to higher CRP level, while the CRP–LDL-C ratio may be a potential risk prediction factor.

## 1. Introduction

What is already known on this topic—A low LDL level is regarded as a protective factor in ACS, while some studies show patients with lower LDL to have a poorer prognosis. Few researchers have studied the role of LDL and inflammation in statin-null patients until now.

What this study adds—We found that low LDL concentrations were related to more short-term and long-term outcomes in statin-null ACS patients and tended to have higher hs-CRP levels. The CRP–LDL ratio has good predictive ability on short-term and long-term outcomes. More attention and aggressive treatments should be added to those spontaneously very low LDL ACS patients with high CRP in clinical practice.

Currently, ischemic heart disease is the leading cause of death, causing heart failure and cardiac death [1]. It imposes heavy burdens on the affected families and society [2]. Several risk factors have been identified during the development and progression of acute coronary syndrome (ACS). Among these factors, lipid level is considered to be one of the most important predictive factors for the prognosis of ACS, and a high lipid level is related to poor prognosis [3]. Low-density lipoprotein (LDL) is a key lipoprotein involved in cholesterol transport [4,5]. It carries cholesterol, which is then measured as low-density lipoprotein cholesterol (LDL-C) in plasma. Our study assessed LDL-C levels and their association with clinical endpoints. Epidemiological studies have demonstrated the contribution of LDL-C-lowering therapy to better outcomes in patients with ACS, which further supported the concept of “the lower the better” [6,7,8]. However, in recent years, several cohort studies have shown associations between low on-admission LDL-C levels and increased mortality in ACS patients [9,10]. This phenomenon is known as the “Lipid paradox”. Until now, the complex role of LDL-C in the progression of ACS remains unclear.

Recent studies imply that lipid-driven inflammation is important during the occurrence and development of atherosclerosis [11]. LDL-C adheres to the vascular wall and activates the downstream inflammatory response [12]. Oxidized LDL induces pyroptosis and inflammation mediated by GSDME [13]. However, the statin therapy history of ACS patients may have complex effects on spontaneous LDL-C levels and anti-inflammation effects independent of lowering the LDL-C. Few studies explored the inflammation status in low-LDL-C ACS patients without statin therapy. High-sensitivity C-reactive protein (hs-CRP) is a common marker of acute inflammation reaction, which is relevant to cardiovascular outcomes [14,15]. Thus, our research aimed to explore the association between CRP and LDL-C levels in the statin-null ACS population.

## 2. Materials and Methods

### 2.1. Study Population

From 1 January 2010 to 31 December 2014, the complete clinical data of 2493 consecutive patients admitted to the cardiac care unit of the Department of Cardiology in Shanghai Renji Hospital with an accurate discharge diagnosis of ACS, including ST-elevation myocardial infarction (STEMI) and non-ST-segment elevation acute coronary syndrome (NSTE-ACS), was subjected to careful screening.

We excluded patients reporting home statin use or for whom information on pre-admission statin prescriptions was not available. To reduce bias and more accurately detect the effect of LDL-C, the on-admission cholesterol profile was strictly limited to within the first 24 h after admission. Patients who had been transferred from other hospitals more than 24 h after the most recent ischemic symptoms, before the initial presentation, and whose current admission was for repeat angiography or selective percutaneous coronary intervention (PCI) for the last ischemic events were also excluded.

Among the total of 2493 patients, 40 patients were excluded due to missing information on pre-hospital statin use, 148 patients were excluded due to the absence of a lipid profile within 24 h after admission, 255 patients were excluded due to transfer-time criteria and repeat angiography or selective PCI, and 168 patients were excluded for self-reported home statin use. Thus, a total of 1882 patients were ultimately included in the assessment. Among these patients, 1009 of them had the hs-CRP test on-admission and were further analyzed. The patients were divided into four groups according to their initial LDL-C level: very low, <70 mg/dL; low, 70–99 mg/dL; high, 100–129 mg/dL and very high, ≥130 mg/dL. In addition, hs-CRP ≥ 2 mg/L was defined as a high-CRP group while hs-CRP < 2 mg/L was defined as a low-CRP group according to multiple large-scale clinical studies on myocardial infarction [16,17,18].

### 2.2. Patient and Public Involvement

Patients, investigators, and statisticians were involved in the design and management of this research. The comprehensible language was used to obtain the patients’ understanding. To improve the cooperation and satisfaction degree of patients, we received suggestions from several patient representatives about follow-up plans. Participants will be informed of the main results of this study.

### 2.3. Data Collection

For all the eligible patients, information on demographics, medical history, clinical characteristics, therapeutic medication, and procedural intervention was extracted from the electronic medical records system of the hospital. In-hospital events and long-term follow-ups were determined from hospital discharge records or by telephone interviews, and were verified by consulting the Social Security Death Certificate Registries.

### 2.4. Statistical Analysis

Statistical analyses to identify risk factors were performed using SPSS19.0 for Windows (SPSS, Chicago, IL, USA). Categorical variables were compared with the χ^2^ test. α continuous variables were compared with the Student’s -t test. Survival curves were produced with the Kaplan–Meier method and compared with the log-rank test. Cox regression analysis was used for multivariate analyses via a stepwise selection process. Age, sex, diabetes mellitus history, PCI surgery, in-hospital treatments, and medications (unfractionated heparin, low molecular weight heparin, beta blocker, ACEI/ARB, statin, and high-dose statins) were included in the model. ROC curves were used to evaluate the prediction ability of the in-hospital and long-term outcomes. *p* < 0.05 was considered statistically significant.

## 3. Results

**Figure 1:**
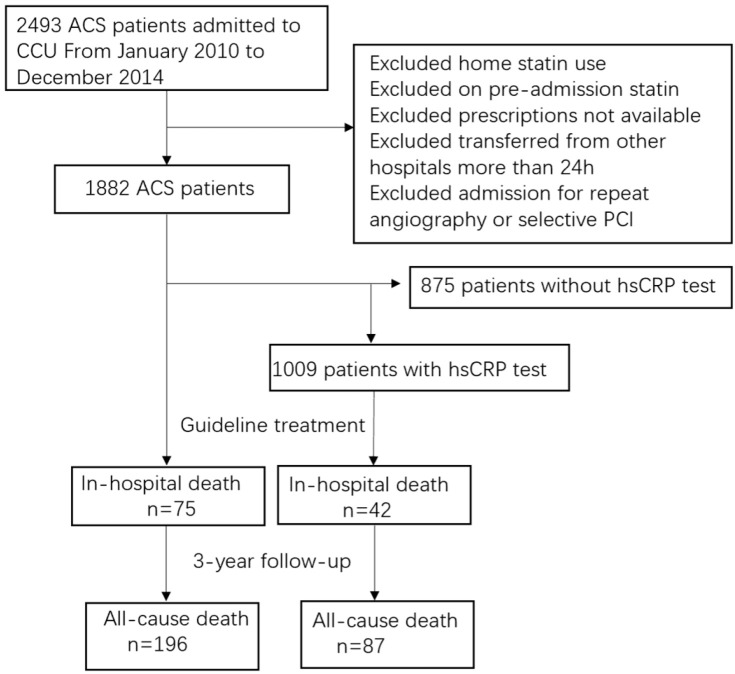
The study flowchart.

In 2493 patients, a total of 1882 statin-null patients with ACS were included in the study. The hs-CRP tests were detected in 1009 of them. After guideline treatment, in-hospital death occurred in 42 patients and long-term deaths occurred in 87 patients. The flow chart of this study is shown in Figure 1. A total of 1714 patients (94.9%) completed the 3-year follow-up. The median follow-up time was 32 months (IQR: 17–47).

### 3.1. Baseline Characteristics in Statin-Null ACS Patients with Different LDL-C Level

The baseline characteristics of all the enrolled statin-null patients were summarized in Table 1. The median age of the population was 62 years, and 20.4% of patients were female. We divided the 1882 patients into 4 groups according to their on-admission LDL-C level: very low, <70 mg/dL (N = 144); low, 70–99 mg/dL (N = 551); high, 100–129 mg/dL (N = 639); and very high, ≥130 mg/dL (N = 548). Over one-third of the patients had an initial LDL-C concentration lower than 100 mg/dL. Patients who presented with lower LDL-C levels were more likely to be older (median ages of 66, 63, 62, and 62 years, *p* = 0.004), male (84.7%, 83.8%, 80.1%, and 73.5%, *p* < 0.001), and had a lower BMI index (23.9, 24.1, 24.5, and 24.7). However, there was little difference among the four groups in the history of prior myocardial infarction (MI) (7.6%, 3.8%, 3.1%, and 3.1%, *p* = 0.058), prior PCI (6.9%, 4.7%, 3.0%, and 3.3%, *p* = 0.086) and prior heart failure (4.2%, 2.2%, 1.4%, and 1.3%, *p* = 0.094).

In the treatment and procedural characteristics, we found that statin-null ACS patients with lower initial LDL-C levels were less likely to receive PCI therapy (Table 1, *p* < 0.001) during the procedure. The rate of early prescription (within 24 h) of guideline-recommended medications, beta-blockers (*p* = 0.001), angiotensin-converting enzyme inhibitors (ACEI)/angiotensin II receptor blockers (ARB) (*p* = 0.001), and statins (*p* = 0.003) decreased in the lower LDL-C groups, while the rate of aspirin and intensive statin did not show any difference (Table 1). Similarly, discharge prescriptions of guideline-recommended medications (aspirin, clopidogrel, beta-blockers, ACEI/ARB, statins, and intensive statins) were less frequent in the lower LDL-C groups (Supplementary Table S1).

### 3.2. LDL-C Paradox in the Prognosis of Statin-Null ACS Patients

**Figure 2:**
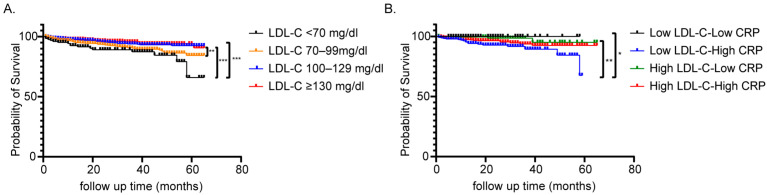
Kaplan–Meier analysis of overall survival in ACS patients. (**A**) Kaplan–Meier analysis of overall survival in 1882 ACS patients by different LDL-C level. (**B**) Kaplan–Meier analysis of overall survival in 1009 ACS patients by different LDL-C and CRP level. *: *p*< 0.05; **: *p*< 0.01; ***: *p*<0.001.

To evaluate the incidence of short-term and long-term outcomes in ACS patients with different initial LDL-C levels, we compared the in-hospital mortality, in-hospital MACE, and long-term mortality in patients with different initial LDL-C levels. As shown in Table 2, we found that patients with very low and low LDL-C levels had higher in-hospital mortality compared to patients with high and very high LDL-C levels (9.7%, 4.5%, 2.7%, 3.5%, *p* = 0.001). Similarly, lower LDL-C level patients also had more in-hospital MACE events (38.9%, 42.3%, 34.3%, 36.7%, *p* = 0.037) and long-term mortality (20.8%, 13.1%, 8.0%, 7.8%, *p* < 0.001). To further understand the long-term survival of patients in each LDL-C group, Kaplan–Meier survival curves were plotted and the statistical differences were analyzed through log-rank analysis. We found that the probability of survival was significantly lower in the very low LDL-C group compared to the high LDL-C group (Figure 2A, *p* < 0.001) or the very high LDL-C group (Figure 2A, *p* < 0.001). Moreover, patients with low LDL-C levels also had a poorer probability of survival compared with patients with very high LDL-C levels (Figure 2A, *p* = 0.005). All these results showed that the ACS patients with low LDL-C levels had poorer short-term and long-term prognoses, which was contrary to our previous lipid regulation belief that lower LDL-C level is better. Then, Cox’s regression analysis was used for the multivariate analyses as implemented with a stepwise selection process (Table 3). Multivariate analysis was performed while accounting for baseline differences in demographics, additional risk factors, and in-hospital treatment. The results showed that very low LDL-C (<70 mg/dL) (HR = 2.09, 95% CI 1.30–3.36) was associated with significantly higher mortality than LDL ≥ 130 mg/dL (Table 3). Age, diabetes, diagnosis of STEMI, and presentation of heart failure were independent risk factors of long-term survival. Patients who underwent PCI therapy had a lower long-term mortality than those treated with conservative therapy. The early prescription of ACEI/ARB and statin in the first 24 h contributed to better survival.

### 3.3. Heterogeneity of CRP Level in Low LDL-C Group Explained for Lipid Paradox

To explore the mechanism of the LDL-C paradox, we further divided the patients with lower LDL-C levels or higher LDL-C levels into different subgroups according to the inflammatory indicators hs-CRP. First, we compared the baseline difference between patients with or without hs-CRP tests to understand the representation of these 1009 patients with hs-CRP tests. As shown in Supplementary Table S2, most of the characteristics were the same except for current smoking rate, total cholesterol level, high-density lipoprotein cholesterol (HDL-C), LDL-C level, peak CK level, in-hospital ACEI/ARB use, and in-hospital intensive statin use. Fewer patients were diagnosed as STEMI in the patients with hs-CRP tests (65.9% vs. 70.9%, *p* = 0.020). Then, these 1009 patients with hs-CRP were further divided into low LDL-C low CRP (LDL-C < 100 mg/dL, hs-CRP < 2 mg/L), low LDL-C high CRP (LDL < 100 mg/dL, hs-CRP ≥ 2 mg/L), high LDL low CRP (LDL ≥ 100 mg/dL, hs-CRP < 2 mg/L), high LDL-C high CRP groups (LDL-C ≥ 100 mg/dL, hs-CRP ≥ 2 mg/L). We found that low LDL-C high CRP patients were older (65.3 vs. 60.8, *p* = 0.002) and had less statin/intensive statin use (statin: 91.9% vs. 98.8, *p* = 0.025; intensive statin: 9.6% vs. 17.9%, *p* = 0.041) compared with low LDL-C low CRP patients (Supplementary Table S3). High LDL-C high CRP patients had higher hypertension history (66.4% vs. 53.9%, *p* = 0.002), prior MI history (4.3% vs. 1.0%, *p* = 0.026) and prior heart failure history (2.6% vs. 0.0%, *p* = 0.044) compared with high LDL-C low CRP patients (Supplementary Table S3). Lower HDL-C level (1.16 vs. 1.23, *p* = 0.005) was found in high LDL-C high CRP patients (Supplementary Table S3).

Interestingly, we found the patients with low LDL-C high CRP had significantly higher in-hospital mortality (7.7% vs. 1.2%, *p* = 0.030) and in-hospital MACE (46.2% vs. 22.6%, *p* < 0.001) compared with the low LDL-C low CRP group (Table 4). Similarly, patients with low LDL-C high CRP also had significantly higher long-term mortality (15.5% vs. 1.2%, *p* = 0.001) compared with the low LDL-C low CRP group (Table 4). These results implied that inflammation status predicted the short-term and long-term outcomes in low LDL-C patients, which might explain the LDL-C paradox. In high LDL-C level patients, high hs-CRP levels were also related to high in-hospital mortality (4.3% vs. 0.5%, *p* = 0.009, Table 4), in-hospital MACE (35.8% vs. 23.0%, *p* < 0.001, Table 4) and long-term mortality (9.4% vs. 2.6%, *p* = 0.002, Table 4). Kaplan–Meier survival curves of these four groups indicated that low LDL-C high CRP patients had a poorer long-term prognosis compared to low LDL-C low CRP patients and high LDL-C low CRP patients (*p* = 0.023, *p* = 0.003, Figure 2B).

### 3.4. The Predictive Ability of the CRP–LDL-C Ratio in Prognosis

**Figure 3:**
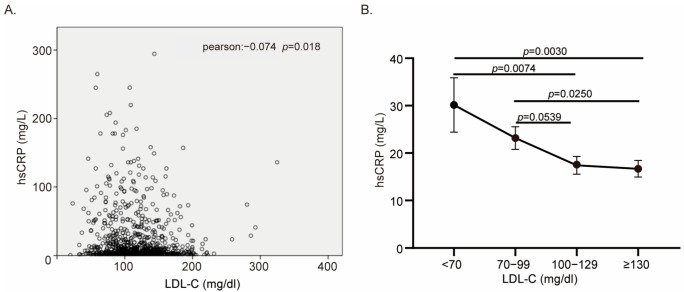
The correlation between the LDL-C and CRP level. (**A**) The scatter plot of LDL-C level and hs-CRP level. (Pearson: −0.074, *p* = 0.018); (**B**) the average hs-CRP level in different grade of LDL-C groups. Data were shown as mean (black dot) and SEM (black lines).

**Figure 4:**
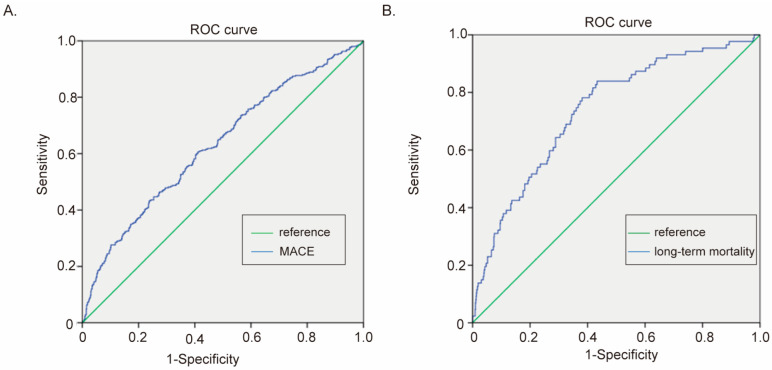
The prediction of prognosis of CRP–LDL-C ratio in 1882 ACS patients. (**A**) ROC curve of CRP–LDL-C ratio in the prediction of in-hospital MACE. AUC: 0.630, 95% CI (0.593–0.666), *p* < 0.001. (**B**) ROC curve of CRP–LDL-C ratio in the prediction of long-term mortality. AUC: 0.738, 95% CI (0.684–0.792), *p* < 0.001.

To figure out the relationship between hs-CRP and LDL-C levels, the scatter plot of hs-CRP and LDL-C was drawn (Figure 3A). The levels of hs-CRP and LDL-C showed a weak negative correlation, which meant that patients with lower LDL-C levels tended to have higher hs-CRP levels (Pearson: −0.074, *p* = 0.018, Figure 3A). Then, we calculated the average hs-CRP level in different LDL-C level groups (very low, low, high, and very high LDL-C groups). As shown in Figure 3B, the mean value of hs-CRP in very low, low, high, and very high LDL-C groups is 30.15, 23.1, 17.42, and 16.70 mg/L, respectively. The hs-CRP level in very low LDL-C patients was significantly higher than it was in high LDL-C patients (*p* = 0.0074, Figure 3B) and very high LDL-C patients (*p* = 0.003, Figure 3B). Similarly, the hs-CRP level in low LDL-C patients was also higher than it was in high LDL-C patients (*p* = 0.0539, Figure 3B) and very high LDL-C patients (*p* = 0.025, Figure 3B). Thus, we calculated the CRP–LDL-C ratio of every patient. The ratio of CRP–LDL-C had a great prediction ability in the in-hospital MACE (AUC under ROC curve: 0.630, 95% CI: 0.593–0.666, *p* < 0.001, Figure 4A) and long-term mortality (AUC under ROC curve:0.738, 95% CI: 0.684–0.792, *p* < 0.001, Figure 4B).

## 4. Discussion

In the present study, about 37% of patients were divided into the very low and low LDL-C group, which indicated that the proportion of spontaneously low LDL-C was high in statin-null ACS patients. While 29.1% of patients were divided into a very high LDL-C group. Similarly, in the CRUSADE Registry, which excluded home statin-used patients, 35.3% of patients had very low and low LDL-C concentrations; the very high group accounted for nearly one-third of the population [19]. LDL-C lowering has been reported as an acute phase response to myocardial infarction [20,21,22,23]. However, this phenomenon often occurs over several days rather than in the first 24 h. Our lipid profiles were obtained within the first 24 h after admission; thus, an acute change in LDL-C level is unlikely to be responsible for the differences observed in the present study.

The Honolulu Heart Program first reported the relationship between low cholesterol and high mortality in the 20-year follow-up of lipid concentration in the elderly [24]. Subsequently, several studies indicated that low LDL-C was associated with worse outcomes in patients with STEMI, NSTE-ACS, and heart failure [9,19,25,26]. Patients with very low LDL-C who were treated with statins have been found to have better outcomes than corresponding patients who were not treated with statins despite the low cholesterol level [26,27]. Until now, the mechanism of the “LDL-C paradox” has been controversial. The most common concept is that it is related to confounding factors associated with survival. The KAMIR Registry found evidence of this paradox, but after adjusting for baseline characteristics, LDL-C level was no longer an independent predictor of mortality [28]. Patients with low LDL-C in the CRUSADE Registry were older than patients with high LDL-C and had more comorbidities, including hypertension, diabetes, and heart failure [19]. In our study, patients in the lower LDL-C categories also had a greater burden of risk factors. Nevertheless, the prognostic effect of spontaneously very low LDL-C remained valid after adjustment for other clinical characteristics. Unlike KAMIR, the present study excluded patients with reported pre-admission statin therapy, allowing the effect of initial LDL-C level without the pleiotropic function of statins to be determined. In addition, in the Münster Heart Study, the increase in mortality at low levels of LDL-C was only observed in smokers and was explained by an increase in smoking-related cancer deaths [20]. However, in the present cohort, there was no difference in current smoking among the LDL-C groups. An age-dependent reduction in LDL-C was observed in a previous study, which used to be considered an effect of poor health status [22]. The multivariate analyses of the present study revealed the independent prognostic values of age and spontaneously very low LDL-C levels. Thus, these two factors play important roles in the development and onset of ACS.

In terms of drug treatment, our patients with lower LDL-C levels tended to be elder and had more comorbidities, which may have contributed to poorer drug tolerance and medication adherence during treatment. In our database, individuals with lower LDL-C levels also had lower rates of PCI therapy and medication use. Similarly, Yang et al. reported that patients with lower LDL-C levels received less PCI therapy and had lower usage rates of statins, oral anticoagulants, beta-blockers, and ACEI/ARBs [29]. Treatment decisions are influenced by various factors, including socioeconomic status, patient compliance, and disease assessment. Further research will be targeted at including those confounding factors. The localization of myocardial infarction should also be analyzed to better understand the characteristics of coronary angiography in those statin-naïve patients, which may influence treatment decisions. Our patients benefited from early initiation of ACEI/ARB and statin, but early prescription of beta-blockers did not contribute to higher survival. The restricted use of beta-blockers in the acute phase might explain this finding. Many of the patients were unstable and could not tolerate this medication, especially in the first 24 h. The protective effect of beta-blockers is more likely due to their early titration and long-term administration [30,31], which was not evaluated in the current study. In the present study, low LDL-C reduced the rate of optimal medical therapy at discharge, particularly the prescription of statins, with the rate ranging from 88.3% to 97.6%. Thus, further studies and improvements to standard treatments in this special ACS population are needed.

Due to the importance of anti-inflammatory therapy and lipid-lowering therapy in AMI, we tried to further figure out the role of inflammation in the “LDL-C paradox”. The study reported that on-treatment CRP but not LDL-C was correlated with outcomes under 24 months of potent statin therapy [32]. Another study investigated the combined utility of hemoglobin level and neutrophil-to-lymphocyte ratio in patients with STEMI undergoing primary PCI [33]. These findings indicated that inflammation might be an important driver of residual cardiovascular risk in patients with coronary artery disease despite aggressive statin therapy. In the subgroup analysis of the present cohort, low LDL-C high CRP group patients had the highest short-term and long-term mortality. Similarly, the newest collaborative analysis of three randomized trials also found that CRP level can predict cardiovascular events [18]. In addition, they found that the predictive value of residual cholesterol risk was neutral compared with residual inflammatory risk [18] which was different from our results. Compared with our cohort, they enrolled high atherosclerotic disease risk patients with current statin treatment, while our study enrolled ACS patients without statin treatment history, which may contribute to the different conclusions. Thus, our study was the first to investigate the relationship between CRP and LDL-C in statin-null ACS patients. The severe inflammation status might be a mechanism contributing to the poor prognosis of patients with spontaneously very low LDL-C levels. We found that spontaneously very low LDL-C levels were associated with a higher probability of inflammation, which may be due to the function of cholesterol in the acute state. Since statins have anti-inflammatory effects, primary and secondary prevention strategies in this unique population require further study.

## 5. Conclusions

In patients with ACS, spontaneously low LDL-C levels on-admission are independently associated with high short-term and long-term mortality, suggesting that improved treatments are needed in this unique population. Spontaneously lower LDL-C patients tended to have higher hs-CRP levels, which could increase the short-term and long-term events. The CRP–LDL-C ratio may be a potential method for predicting the ACS prognosis.

## 6. Limitations

This study has some limitations. First, the retrospective study design might have led to selection bias, treatment bias, and unequal distributions of baseline comorbidities among the groups. Second, although we performed multivariate analysis and included as many baseline variables as possible, we could not account for all possible confounding characteristics and treatment effects. For example, the lack of cancer and cachexia information may induce bias. However, BMI is also a common indicator of cachexia [34]. BMI < 20 kg/m^2^ is sufficient for cachexia if weight loss cannot be recorded [35]. The mean (SD) BMI of our patients was 24.4 (3.2) kg/m^2^, suggesting that cachexia was uncommon in our cohort. For another, our study cannot include all the chronic diseases. Thus, we have included chronic diseases associated with a high risk of heart disease such as hypertension, diabetes, heart failure history, stroke history, and MI history to reduce bias. Other chronic diseases such as connective diseases, COPD, hematological or oncological diseases, are also becoming a hotspot in the prognosis studies of cardiovascular diseases. Further studies are needed to make the conclusion more accurate. Third, we recorded only from discharge information, while information on therapies during follow-up, whose effects on outcomes are not clear, was not available. Additionally, our patient cohort was enrolled between 2010 and 2014, which may introduce treatment bias due to subsequent advances in therapy. Finally, although we compared the baseline between patients with or without hs-CRP tests, the prognostic value of the CRP–LDL-C ratio requires validation with a larger data set. The current conclusions were limited to the Chinese population. These conclusions need to be further confirmed in a larger cohort of patients with a uniform therapeutic approach in subsequent studies.

## Figures and Tables

**Table 1:** Baseline, treatment, and outcome characteristics of the study population by LDL-C level.

	All Patients (N = 1882)	LDL-C Level at Presentation (mg/dL)				*p* Value
		<70	70–99	100–129	≥130	
		(N = 144)	(N = 551)	(N = 639)	(N = 548)	
Demographics						
Age, median (IQR), y	62 (55–72)	66 (57–75)	63 (56–73)	62 (56–72)	62 (54–71)	0.004
Female sex, *n* (%)	383 (20.4)	22 (15.3)	89 (16.2)	127 (19.9)	145 (26.5)	<0.001
Cardiac risk factors, *n* (%)						
Current smoker	845 (44.9)	69 (47.9)	256 (46.5)	270 (42.3)	250 (45.6)	0.392
Hypertension	1161 (61.7)	86 (59.7)	336 (61.0)	399 (62.4)	340 (62.0)	0.911
Diabetes mellitus	462 (24.5)	43 (29.9)	151 (27.4)	135 (21.1)	133 (24.3)	0.034
Family history of CAD	91 (4.8)	6 (4.2)	24 (4.4)	35 (5.5)	26 (4.7)	0.802
Prior stroke	217 (11.5)	18 (12.5)	70 (12.7)	74 (11.6)	55 (10.0)	0.556
Prior MI	69 (3.7)	11 (7.6)	21 (3.8)	20 (3.1)	17 (3.1)	0.058
Prior PCI	73 (3.9)	10 (6.9)	26 (4.7)	19 (3.0)	18 (3.3)	0.086
Prior heart failure	34 (1.8)	6 (4.2)	12 (2.2)	9 (1.4)	7 (1.3)	0.094
BMI (kg/m^2^)	24.4 (3.2)	23.9 (3.4)	24.1 (3.2)	24.5 (3.1)	24.7 (3.2)	0.037
Diagnosis						0.230
STEMI	1284 (68.2)	91 (63.2)	363 (65.9)	447 (70.0)	383 (69.9)	
NSTE-ACS	598 (31.8)	53 (8.9)	188 (31.4)	192 (32.1)	165 (27.6)	
In-hospital treatment						
Cardiac catheterization	1783 (94.7)	131 (91.0)	518 (94.0)	614 (96.1)	520 (94.9)	0.071
PCI	1640 (87.1)	115 (79.9)	457 (82.9)	574 (89.8)	494 (90.1)	<0.001
Thrombolytic therapy	170 (9.0)	9 (6.3)	41 (7.4)	66 (10.3)	54 (9.9)	0.186
Heparin administration						
IV unfractionated	1417 (75.3)	106 (73.6)	391 (71.0)	491 (76.8)	429 (78.3)	0.026
Low molecular weight	1289 (68.5)	82 (56.9)	398 (72.2)	445 (69.6)	364 (66.4)	0.003
Medications w/in 24 hours						
Aspirin	1863 (99.0)	140 (97.2)	546 (99.1)	634 (99.2)	543 (99.1)	0.318
Beta-blocker	937 (49.8)	55 (38.2)	253 (45.9)	330 (51.6)	299 (54.6)	0.001
ACEI/ARB	1142 (60.7)	66 (45.8)	329 (59.7)	413 (64.6)	334 (60.9)	0.001
Statin	1817 (96.5)	133 (92.4)	525 (95.3)	621 (97.2)	538 (98.2)	0.003
Intensive statin	104 (19.0)	16 (11.1)	82 (14.9)	105 (16.4)	104 (19.0)	0.088

Continuous values are expressed as the mean ± standard deviation or median (with 25th and 75th percentiles); categorical values are expressed as total number and proportion of the global population (in parentheses). CAD, coronary artery disease; MI, myocardial infarction; PCI, percutaneous coronary intervention; STEMI, ST-segment elevation myocardial infarction; NSTE-ACS, non-ST-segment elevation acute coronary syndrome; IV, intravenous; ACEI, angiotensin converting enzyme inhibitors; ARB, angiotensin II receptor blockers; BMI, body mass index.

**Table 2:** The short-term and long-term outcomes between different LDL-C patients.

	LDL-C Level at Presentation (mg/dL)				*p* Value
	<70 (N = 144)	70–99 (N = 551)	100–129 (N = 639)	≥130 (N = 548)	
In-hospital mortality, *n* (%)	14 (9.7)	25 (4.5)	17 (2.7)	19 (3.5)	0.001
In-hospital MACE, *n* (%)	56 (38.9)	233 (42.3)	219 (34.3)	201 (36.7)	0.037
Long-term mortality, *n* (%)	30 (20.8)	72 (13.1)	51 (8.0)	43 (7.8)	<0.001

Values are expressed as numbers (percentage). LDL-C: low-density lipoprotein cholesterol; MACE: major adverse cardiovascular events.

**Table 3:** Cox’s proportional hazard regression model of long-term mortality in 1882 ACS patients.

Parameter	*p* Value	HR	95% CI	
			Lower	Upper
On-admission LDL-C level				
<70 mg/dL	0.002	2.09	1.30	3.36
70–89 mg/dL	0.169	1.31	0.89	1.91
90–129 mg/dL	0.382	0.83	0.55	1.26
≥130 mg/dL		Reference		
Demographics				
Age	<0.001	1.07	1.05	1.09
Cardiac risk factors				
Diabetes mellitus	0.008	1.50	1.11	2.02
Clinical presentation				
Heart failure	<0.001	2.29	1.69	3.11
Diagnosis				
STEMI	0.007	1.59	1.14	2.23
In-hospital treatment				
PCI	<0.001	0.28	0.21	0.38
ACEI/ARB in first 24 h	0.003	0.65	0.48	0.87
Statin in first 24 h	0.001	0.42	0.25	0.70

LDL-C, low-density lipoprotein cholesterol; STEMI, ST-segment elevation myocardial infarction; PCI, percutaneous coronary intervention; ACEI, angiotensin converting enzyme inhibitors; ARB, angiotensin II receptor blockers. HR: hazard ratio.

**Table 4:** The short-term and long-term outcomes between different CRP-level groups in spontaneously low LDL patients.

	Low LDL-C < 100		*p* Value	High LDL-C ≥ 100		*p* Value
	Low CRP (<2)	High CRP (≥2)		Low CRP (<2)	High CRP (≥2)	
In-hospital mortality	1 (1.2)	20 (7.7)	0.030	1 (0.5)	20 (4.3)	0.009
In-hospital MACE	19 (22.6)	120 (46.2)	<0.001	47 (23.0)	165 (35.8)	<0.001
Long-term mortality	1 (1.2)	39 (15.5)	0.001	5 (2.6)	42 (9.4)	0.002

LDL-C, low-density lipoprotein cholesterol; CRP, c-reactive protein; MACE, major adverse cardiovascular events.

## Data Availability

The datasets used and analyzed during the current study are available from the corresponding author on reasonable request.

## Supplementary Materials

The following supporting information can be downloaded at: https://www.mdpi.com/article/10.3390/biomedicines13071534/s1. Supplementary Table S1. Discharge treatment by LDL level, Supplementary Table S2. The difference of baseline characteristics between patients with or without hs-CRP tests, Supplementary Table S3. The baseline characteristics of the patients with Low and High CRP level.

## Abbreviations

The following abbreviations are used in this manuscript:

LDL-C	Low-density lipoprotein cholesterol
ACS	Acute coronary syndrome
hs-CRP	High-sensitivity C-reactive protein
STEMI	ST-elevation myocardial infarction
NSTE-ACS	Non-ST-segment elevation acute coronary syndrome
PCI	Percutaneous coronary intervention
HDL-C	High-density lipoprotein cholesterol
ACEI	Angiotensin-converting enzyme inhibitors
ARB	Angiotensin II receptor blockers

## References

[1] Nowbar A.N., Gitto M., Howard J.P., Francis D.P., Al-Lamee R. (2019). Mortality From Ischemic Heart Disease. Circ. Cardiovasc. Qual. Outcomes.

[2] Gheorghe A., Griffiths U., Murphy A., Legido-Quigley H., Lamptey P., Perel P. (2018). The economic burden of cardiovascular disease and hypertension in low- and middle-income countries: A systematic review. BMC Public Health.

[3] Yang Q., Sun D., Pei C., Zeng Y., Wang Z., Li Z., Hao Y., Song X., Li Y., Liu G. (2021). LDL cholesterol levels and in-hospital bleeding in patients on high-intensity antithrombotic therapy: Findings from the CCC-ACS project. Eur. Heart J..

[4] Mineo C. (2020). Lipoprotein receptor signalling in atherosclerosis. Cardiovasc. Res..

[5] Getz G.S., Reardon C.A. (2018). Apoprotein E and Reverse Cholesterol Transport. Int. J. Mol. Sci..

[6] Averna M., Banach M., Bruckert E., Drexel H., Farnier M., Gaita D., Magni P., März W., Masana L., Mello E.S.A. (2021). Practical guidance for combination lipid-modifying therapy in high- and very-high-risk patients: A statement from a European Atherosclerosis Society Task Force. Atherosclerosis.

[7] Baigent C., Blackwell L., Emberson J., Holland L.E., Reith C., Bhala N., Peto R., Barnes E.H., Keech A., Simes J. (2010). Efficacy and safety of more intensive lowering of LDL cholesterol: A meta-analysis of data from 170,000 participants in 26 randomised trials. Lancet.

[8] Claessen B.E., Guedeney P., Gibson C.M., Angiolillo D.J., Cao D., Lepor N., Mehran R. (2020). Lipid Management in Patients Presenting with Acute Coronary Syndromes: A Review. J. Am. Heart Assoc..

[9] Cheng K.H., Chu C.S., Lin T.H., Lee K.T., Sheu S.H., Lai W.T. (2015). Lipid paradox in acute myocardial infarction-the association with 30-day in-hospital mortality. Crit. Care Med..

[10] Al-Mallah M.H., Hatahet H., Cavalcante J.L., Khanal S. (2009). Low admission LDL-cholesterol is associated with increased 3-year all-cause mortality in patients with non ST segment elevation myocardial infarction. Cardiol. J..

[11] Bäck M., Yurdagul A., Tabas I., Öörni K., Kovanen P.T. (2019). Inflammation and its resolution in atherosclerosis: Mediators and therapeutic opportunities. Nat. Rev. Cardiol..

[12] Libby P., Ridker P.M., Hansson G.K. (2011). Progress and challenges in translating the biology of atherosclerosis. Nature.

[13] Wei Y., Lan B., Zheng T., Yang L., Zhang X., Cheng L., Tuerhongjiang G., Yuan Z., Wu Y. (2023). GSDME-mediated pyroptosis promotes the progression and associated inflammation of atherosclerosis. Nat. Commun..

[14] Denegri A., Boriani G. (2021). High Sensitivity C-reactive Protein (hsCRP) and its Implications in Cardiovascular Outcomes. Curr. Pharm. Des..

[15] van den Berg V.J., Umans V., Brankovic M., Oemrawsingh R.M., Asselbergs F.W., van der Harst P., Hoefer I.E., Kietselaer B., Crijns H., Lenderink T. (2020). Stabilization patterns and variability of hs-CRP, NT-proBNP and ST2 during 1 year after acute coronary syndrome admission: Results of the BIOMArCS study. Clin. Chem. Lab. Med..

[16] Ridker P.M., Everett B.M., Thuren T., MacFadyen J.G., Chang W.H., Ballantyne C., Fonseca F., Nicolau J., Koenig W., Anker S.D. (2017). Antiinflammatory Therapy with Canakinumab for Atherosclerotic Disease. N. Engl. J. Med..

[17] Carrero J.J., Andersson Franko M., Obergfell A., Gabrielsen A., Jernberg T. (2019). hsCRP Level and the Risk of Death or Recurrent Cardiovascular Events in Patients With Myocardial Infarction: A Healthcare-Based Study. J. Am. Heart Assoc..

[18] Ridker P.M., Bhatt D.L., Pradhan A.D., Glynn R.J., MacFadyen J.G., Nissen S.E. (2023). Inflammation and cholesterol as predictors of cardiovascular events among patients receiving statin therapy: A collaborative analysis of three randomised trials. Lancet.

[19] O’Brien E.C., Simon D.N., Roe M.T., Wang T.Y., Peterson E.D., Alexander K.P. (2015). Statin Treatment by Low-Density Lipoprotein Cholesterol Levels in Patients with Non-ST-Segment Elevation Myocardial Infarction/Unstable Angina Pectoris (from the CRUSADE Registry). Am. J. Cardiol..

[20] Cullen P., Schulte H., Assmann G. (1997). The Münster Heart Study (PROCAM): Total mortality in middle-aged men is increased at low total and LDL cholesterol concentrations in smokers but not in nonsmokers. Circulation.

[21] Iseki K., Yamazato M., Tozawa M., Takishita S. (2002). Hypocholesterolemia is a significant predictor of death in a cohort of chronic hemodialysis patients. Kidney Int..

[22] Volpato S., Zuliani G., Guralnik J.M., Palmieri E., Fellin R. (2001). The inverse association between age and cholesterol level among older patients: The role of poor health status. Gerontology.

[23] Volpato S., Leveille S.G., Corti M.C., Harris T.B., Guralnik J.M. (2001). The value of serum albumin and high-density lipoprotein cholesterol in defining mortality risk in older persons with low serum cholesterol. J. Am. Geriatr. Soc..

[24] Schatz I.J., Masaki K., Yano K., Chen R., Rodriguez B.L., Curb J.D. (2001). Cholesterol and all-cause mortality in elderly people from the Honolulu Heart Program: A cohort study. Lancet.

[25] Güder G., Frantz S., Bauersachs J., Allolio B., Wanner C., Koller M.T., Ertl G., Angermann C.E., Störk S. (2009). Reverse epidemiology in systolic and nonsystolic heart failure: Cumulative prognostic benefit of classical cardiovascular risk factors. Circ. Heart Fail..

[26] Miura M., Yamasaki M., Uemura Y., Yoshikawa M., Miyauchi K., Tanaka H., Miyachi H., Yamashita J., Suzuki M., Yamamoto T. (2016). Effect of Statin Treatment and Low-Density Lipoprotein-Cholesterol on Short-Term Mortality in Acute Myocardial Infarction Patients Undergoing Primary Percutaneous Coronary Intervention—Multicenter Registry from Tokyo CCU Network Database. Circ. J..

[27] Lee K.H., Jeong M.H., Kim H.M., Ahn Y., Kim J.H., Chae S.C., Kim Y.J., Hur S.H., Seong I.W., Hong T.J. (2011). Benefit of early statin therapy in patients with acute myocardial infarction who have extremely low low-density lipoprotein cholesterol. J. Am. Coll. Cardiol..

[28] Cho K.H., Jeong M.H., Ahn Y., Kim Y.J., Chae S.C., Hong T.J., Seong I.W., Chae J.K., Kim C.J., Cho M.C. (2010). Low-density lipoprotein cholesterol level in patients with acute myocardial infarction having percutaneous coronary intervention (the cholesterol paradox). Am. J. Cardiol..

[29] Sun H., Li Z., Song X., Liu H., Li Y., Hao Y., Teng T., Liu J., Liu J., Zhao D. (2021). Revisiting the lipid paradox in ST-elevation myocardial infarction in the Chinese population: Findings from the CCC-ACS project. Eur. Heart J. Acute Cardiovasc. Care.

[30] Steg P.G., James S.K., Atar D., Badano L.P., Blömstrom-Lundqvist C., Borger M.A., Di Mario C., Dickstein K., Ducrocq G., Fernandez-Aviles F. (2012). ESC Guidelines for the management of acute myocardial infarction in patients presenting with ST-segment elevation. Eur. Heart J..

[31] Chen Z.M., Pan H.C., Chen Y.P., Peto R., Collins R., Jiang L.X., Xie J.X., Liu L.S. (2005). Early intravenous then oral metoprolol in 45,852 patients with acute myocardial infarction: Randomised placebo-controlled trial. Lancet.

[32] Puri R., Nissen S.E., Libby P., Shao M., Ballantyne C.M., Barter P.J., Chapman M.J., Erbel R., Raichlen J.S., Uno K. (2013). C-reactive protein, but not low-density lipoprotein cholesterol levels, associate with coronary atheroma regression and cardiovascular events after maximally intensive statin therapy. Circulation.

[33] Cho K.H., Jeong M.H., Ahmed K., Hachinohe D., Choi H.S., Chang S.Y., Kim M.C., Hwang S.H., Park K.H., Lee M.G. (2011). Value of early risk stratification using hemoglobin level and neutrophil-to-lymphocyte ratio in patients with ST-elevation myocardial infarction undergoing primary percutaneous coronary intervention. Am. J. Cardiol..

[34] Ueshima J., Inoue T., Saino Y., Kobayashi H., Murotani K., Mori N., Maeda K. (2024). Diagnosis and prevalence of cachexia in Asians: A scoping review. Nutrition.

[35] Evans W.J., Morley J.E., Argilés J., Bales C., Baracos V., Guttridge D., Jatoi A., Kalantar-Zadeh K., Lochs H., Mantovani G. (2008). Cachexia: A new definition. Clin. Nutr..

