# Supplementary material for: Initial Low-Density Lipoprotein Cholesterol and Inflammation Status Predicts Long-Term Mortality in Patients with Acute Coronary Syndrome in the Chinese Population
Source: Biomedicines. 2025 Jun 24;13(7):1534. doi: 10.3390/biomedicines13071534 (PMC12292495; doi:10.3390/biomedicines13071534)
Supplement: Supplementary file 1 [file biomedicines-13-01534-s001.zip › biomedicines-3582399-supplementary.pdf]

Supplementary Table1. Discharge treatment by LDL level

Medications prescribed, n (%)	All Patients N=1788	LDL level at presentation (mg/dL)					
		<70 (N=128)	70-99 (N=521)	100-129 (N=615)	≥130 (N=524)	P Value	P_{trend} Value
Aspirin	1744 (97.5)	122 (95.3)	505 (96.9)	600 (97.6)	517 (98.7)	0.104	<0.001
Clopidogrel	1669 (93.3)	111 (86.7)	484 (92.9)	577 (93.8)	497 (94.8)	0.010	<0.001
Ticagrelor	58 (3.2)	7 (5.5)	9 (1.7)	22 (3.6)	20 (3.8)	0.072	0.392
Nitroglycerin	550 (30.8)	40 (31.3)	166 (31.9)	184 (29.9)	160 (30.5)	0.913	0.958
Beta-blocker	1370 (76.6)	91 (71.1)	371 (71.2)	490 (79.7)	418 (79.8)	0.001	<0.001
ACEI/ARB	1286 (71.9)	75 (58.6)	373 (71.6)	461 (75.0)	377 (71.9)	0.003	0.005
Calcium-channel blocker	146 (8.2)	15 (11.7)	44 (8.4)	45 (7.3)	42 (8.0)	0.421	0.383
Statin	1720 (96.2)	113 (88.3)	496 (95.2)	600 (97.6)	511 (97.5)	<0.001	<0.001
Intensive statin	97 (5.4)	4 (3.1)	15 (2.9)	24 (3.9)	54 (10.3)	<0.001	<0.001

Categorical values were expressed as total number and proportion of the global population (in parentheses). LDL, low-density lipoprotein; ACEI, angiotensin converting enzyme inhibitors; ARB, angiotensin II receptor blockers

Supplementary Table 2. The difference of baseline characteristics between patients with or without hs-CRP tests.

	With hs-CRP test	Without hs-CRP test	P value
**Demographics**			
Age, median (IQR)	63.17	62.94	0.671
Female sex, n (%)	208 (20.6)	175 (20.0)	0.760
**Cardiac risk factors, n (%)**			
Current smoker	501 (49.7)	344 (39.4)	<0.001
Hypertension	635 (62.9)	526 (60.3)	0.233
Diabetes mellitus	252 (25)	210 (24.1)	0.644
Family history of CAD	53 (5.3)	38 (4.4)	0.364
Prior stroke	111 (11.0)	106 (12.1)	0.440
Prior MI	40 (4.0)	29 (3.3)	0.460
Prior PCI	43 (4.3)	30 (3.4)	0.355
Prior heart failure	23 (2.3)	11 (1.3)	0.098

**Diagnosis, n (%)**			
STEMI	655 (65.9)	619 (70.9)	0.020
NSTEMI-ACS	344 (34.1)	254 (29.1)	0.020
**Blood examination, mean (SD)**			
TC (mmol/L)	4.83 (1.2)	4.64 (1.0)	<0.001
TG (mmol/L)	1.57 (1.1)	1.67 (1.3)	0.069
HDL (mmol/L)	1.16 (0.3)	1.12 (0.3)	0.022
LDL (mg/dl)	117.1 (40.4)	111.0 (32.2)	<0.001
CK peak (U/L)	1840.0 (2004.1)	2045.9 (2530.4)	0.049
**In-hospital medications, n (%)**			
Aspirin	1000 (99.1)	863 (98.9)	0.583
Beta-blocker	486 (48.2)	451 (51.7)	0.131
ACEI/ARB	574 (56.9)	568 (65.1)	<0.001
Statin	970 (96.1)	847 (97.0)	0.293
Intensive statin	143 (14.2)	164 (18.8)	0.007

Continuous values are expressed as the mean ± standard deviation; categorical values are expressed as number (percentage). LDL, low-density lipoprotein; CRP, c-reactive protein; CAD, coronary artery disease; MI, myocardial infarction; PCI, percutaneous coronary intervention; STEMI, ST-segment elevation myocardial infarction; NSTE-ACS, non-ST-segment elevation acute coronary syndrome; TC, total cholesterol; TG, total triglyceride; HDL, high-density lipoprotein; CK, creatine kinase; ACEI, angiotensin converting enzyme inhibitors; ARB, angiotensin II receptor blockers.

Supplementary Table 3. The baseline characteristics of the patients with Low and High CRP level.

	Low LDL(<100mg/dl)		P value	High LDL (>100mg/dl)		P value
	Low CRP (<2mg/L)	High CRP (≥2mg/L)		Low CRP (<2mg/L)	High CRP (≥2mg/L)	
**Demographics**						
Age, median (IQR), y	60.8 (11.0)	65.3 (11.9)	0.002	62.4 (10.9)	62.7 (12.2)	0.715
Female sex, n (%)	16 (19.0)	42 (16.2)	0.538	42 (20.6)	108 (23.4)	0.419
**Cardiac risk factors, n (%)**						
Current smoker	42 (50.0)	133 (51.2)	0.854	103 (50.5)	223 (48.4)	0.615
Hypertension	49 (58.3)	170 (65.4)	0.243	110 (53.9)	306 (66.4)	0.002
Diabetes mellitus	21 (25.0)	81 (31.2)	0.283	41 (20.1)	109 (23.6)	0.313
Family history of CAD	7 (8.3)	11 (4.2)	0.236	15 (7.4)	20 (4.3)	0.108
Prior stroke	7 (8.3)	32 (12.3)	0.318	19 (9.3)	53 (11.5)	0.403

Prior MI	4 (4.8)	14 (5.4)	1.000	2 (1.0)	20 (4.3)	0.026
Prior PCI	6 (7.1)	14 (5.4)	0.741	3 (1.5)	20 (4.3)	0.062
Prior heart failure	1 (1.2)	10 (3.8)	0.398	0 (0.0)	12 (2.6)	0.044
**Diagnosis**						
STEMI	46 (54.8)	166 (63.8)	0.137	146 (71.6)	307 (66.6)	0.204
NSTEMI-ACS	38 (45.2)	94 (36.2)	0.137	58 (28.4)	154 (33.4)	0.204
**Blood examination, mean (SD)**						
TC (mmol/L)	3.72 (0.64)	3.78 (0.71)	0.529	5.38 (0.89)	5.38 (1.10)	0.973
TG (mmol/L)	1.38(1.05)	1.39 (1.06)	0.895	1.64 (0.99)	1.68 (1.06)	0.635
HDL (mmol/L)	1.14 (0.28)	1.10 (0.33)	0.297	1.23 (0.30)	1.16 (0.28)	0.005
LDL (mg/dl)	79.0 (17.5)	79.7 (16.4)	0.772	135.5 (27.1)	137.1 (37.9)	0.597
CRP (mg/l)	1.0 (0.5)	32.5 (45.7)	<0.001	1.0 (0.5)	24.2 (37.3)	<0.001
CK peak(U/L)	1458.9 (1713.4)	1531.3 (1889.1)	0.755	2087.7 (2095.0)	1974.0 (2049.2)	0.512
**In-hospital medications, n (%)**						
Aspirin	83 (98.8)	257 (98.8)	1.000	203 (99.5)	457 (99.1)	1.000
Beta-blocker	44 (52.4)	106 (40.8)	0.062	111 (54.4)	225 (48.8)	0.182
ACEI/ARB	49 (58.3)	145 (55.8)	0.680	119 (58.3)	261 (56.6)	0.680
Statin	83 (98.8)	239 (91.9)	0.025	202 (99.0)	446 (96.7)	0.087
Intensive statin	15 (17.9)	25 (9.6)	0.041	28 (13.7)	75 (16.3)	0.403

Continuous values are expressed as the mean ± standard deviation; categorical values are expressed as number (percentage). LDL, low-density lipoprotein; CRP, c-reactive protein; CAD, coronary artery disease; MI, myocardial infarction; PCI, percutaneous coronary intervention; STEMI, ST-segment elevation myocardial infarction; NSTE-ACS, non-ST-segment elevation acute coronary syndrome; TC, total cholesterol; TG, total triglyceride; HDL, high-density lipoprotein; CK, creatine kinase; ACEI, angiotensin converting enzyme inhibitors; ARB, angiotensin II receptor blockers.
